# Drought-Induced Mortality in *Zanthoxylum planispinum* var. *dingtanensis* Is Associated with Sap Flow Dysregulation and Narrow Hydraulic Safety Margins

**DOI:** 10.3390/plants15142145

**Published:** 2026-07-12

**Authors:** Kaiping Li, Zhiying Yang, Yuan Li, Jiaxian Sheng, Meihong Luo

**Affiliations:** 1 College of Architecture and Urban Planning, Guizhou Institute of Technology, Guiyang 550025, China; likaiping3extra@163.com (K.L.); shengjiaxian05@163.com (J.S.); luomeihong1669@163.com (M.L.); 2 School of Geography and Environmental Science, Guizhou Normal University, Guiyang 550025, China; yangggzy0513@163.com; 3 Anshun Agricultural Environment Field Observation and Research Station of the Ministry of Agriculture and Rural Affairs, Anshun 561301, China; 4 School of Karst Science, Guizhou Normal University, Guiyang 550025, China

**Keywords:** drought, sap flow, environmental factors, xylem vulnerability curve, hydraulic safety margin, *Zanthoxylum planispinum*

## Abstract

In the context of global climate change, drought events significantly impact plants, and studying the response of plant sap flow to environmental factors is of great significance for plant protection and sustainable development. This article uses the thermal pulse method to obtain trunk sap flow data, combined with real-time monitoring of environmental factor data, to compare the differences in sap flow characteristics between the subsequently deceased and surviving *Zanthoxylum planispinum* var. *dingtanensis* during a natural drought event. Additionally, the xylem vulnerability curve of *Z. planispinum* was fitted through laboratory measurements to estimate its hydraulic safety margin (HSM). The results showed that: (1) Compared with the surviving individuals, the subsequently deceased *Z. planispinum* exhibited a higher and more fluctuating sap flow rate, maintaining a consistently higher transpiration rate even after the onset of drought. (2) As soil moisture decreased, the sap flow of surviving *Z. planispinum* was promptly constrained by soil water availability and remained at a low, conservative level. In contrast, the sap flow of the deceased individuals continued to be driven by meteorological factors, failing to downregulate transpiration. (3) The xylem vulnerability curve revealed a $P_{50}$ of −2.2 MPa. During the severe drought, the minimum field branch water potential ($\psi_{min}$) dropped to −3.0 MPa, resulting in a negative HSM (HSM = −0.8 MPa). This suggests that the xylem tension severely breached the embolism threshold, likely triggering catastrophic hydraulic failure, which emerged as the primary driver of the observed mortality in *Z. planispinum*. Therefore, for plants with low HSMs, when they are subjected to drought stress accompanied by highly fluctuating sap flow—a condition indicating that water supply cannot match the transpirational demand—timely manual intervention (e.g., supplemental irrigation) may be considered to mitigate hydraulic risks and ensure survival.

## 1. Introduction

Arid and semi-arid regions globally account for approximately 40% of the Earth’s land surface, representing some of the most fragile ecosystems under a changing climate [1]. These regions are predominantly constrained by harsh climatic factors, including erratic precipitation, extreme thermal radiation, and increasingly sustained high vapor pressure deficit (VPD) [2]. The edaphic conditions further exacerbate these climatic stressors, as the soil profiles are typically shallow, poorly structured, deficient in organic nutrients, and characterized by extremely low water retention capacity. Among these vulnerable environments, the karst ecosystems—widely distributed across southwestern China—represent an extreme analogue of arid and semi-arid landscapes [3]. Karst topography is distinctly characterized by a highly heterogeneous “dual soil-rock structure”. The surface soil is remarkably shallow (often <50 cm) and discontinuous, resting upon a highly permeable carbonate bedrock. Consequently, precipitation rapidly leaks into deep underground fissures, epikarst zones, and sinkholes, leaving the topsoil severely desiccated [3]. Even short intervals without rainfall can trigger severe physiological water stress, rendering the karst rocky desertification areas highly susceptible to extreme flash droughts and transforming them into harsh “dry-hot valleys”. To survive and establish in such multi-stressed environments, adapted woody plant species have evolved specialized morphological and physiological trait syndromes. Physiologically, these woody species often develop robust xylem anatomies characterized by high resistance to cavitation—typically indicated by highly negative $P_{50}$ values—which helps prevent lethal hydraulic failure under extreme tension [4]. Morphologically, they may also employ conservative stomatal regulation (isohydric behavior) or develop dimorphic root systems to access isolated subsurface water [5]. Beyond their remarkable physiological resilience, these adapted species hold immense ecological and socioeconomic significance. As pioneer vegetation, they play a crucial role in ecological restoration, significantly contributing to mitigating rocky desertification, conserving soil and water, and stabilizing local microclimates [6]. *Zanthoxylum planispinum* var. *dingtanensis* is a quintessential adapted pioneer species widely distributed in the subtropical karst dry-hot valleys of southwestern China. Due to its remarkable physiological resilience, it serves simultaneously as a critical ecological barrier against soil erosion and a primary cash crop sustaining regional livelihoods. However, the exact physiological thresholds of such robust species when confronted with unprecedented extreme climate events remain poorly understood. In late 2023, an exceptional extreme drought event triggered widespread, catastrophic mortality among *Z. planispinum* plantations in the region. This sudden die-off provides a unique, albeit devastating, opportunity to investigate the physiological limits of drought-adapted species in naturally fragile habitats. Therefore, this study aims to unravel the physiological mechanisms driving this mass mortality. Specifically, we coupled meteorological dynamic indicators (e.g., SESR) with continuous sap flow monitoring and precise xylem vulnerability traits ($P_{50}$, $P_{88}$, and the true Hydraulic Safety Margin (HSM) calculated from $\psi_{min}$). We hypothesized that during extreme flash droughts, the extreme atmospheric evaporative demand would drive the in situ xylem tension of *Z. planispinum* beyond its safety threshold, triggering irreversible and catastrophic hydraulic failure.

## 2. The Study Area and Research Methods

### 2.1. The Study Area and Crop Management

The study area is situated at the Guanling Karst Ecosystem Field Scientific Observation and Research Station in Anshun City, Guizhou Province (105°36′30″–105°46′30″ E, 25°39′13″–25°41′00″ N). It is situated in the middle reaches of the Beipan River basin, which forms part of the Pearl River basin. The area features a plateau–canyon landscape with pronounced topographic relief, with elevations ranging from approximately 485 m at the Beipan River surface to 1250 m at the northern slope crest. Climatically, the region falls within a subtropical dry-hot valley zone, with a mean annual temperature of 18.4 °C and an average annual precipitation of 1100 mm, over 80% of which is concentrated between May and October.

The lithology of the study area is dominated by Middle Triassic dolomitic limestone, with yellow soil and calcareous soil as the primary soil types. Historically unreasonable cultivation and severe human disturbances have led to substantial soil erosion and a high rock outcrop rate, resulting in severe karst rocky desertification. Consequently, the soil layer is extremely thin and discontinuous, with depths ranging only from 30 to 62 cm [7]. Since 1996, the region has been designated as a national demonstration area for rocky desertification control, implementing slope-to-terrace conversion projects as the primary soil and water conservation measure.

Under these harsh, drought-prone conditions, drought-tolerant perennial plants, particularly *Z. planispinum*, are extensively cultivated and have become the pillar economic industry of the local region. Regarding crop management, local farmers implement regular pruning to eliminate apical dominance, maintaining the mature *Z. planispinum* trees (which enter peak production at 6–7 years) at an average height of 2.0–2.5 m with highly consistent crown widths. Crucially, due to the highly fragmented karst topography and poor road accessibility, artificial irrigation is nearly impossible to implement. Therefore, the *Z. planispinum* plantations are entirely rainfed. When natural precipitation fails to meet their high transpirational demand driven by the dry-hot valley climate (foehn effect and intense thermal radiation from exposed bedrock), these plants are exposed to severe drought stress and fatal hydraulic risks.

### 2.2. Research Background

The species *Z. planispinum* is a drought-tolerant and photophilous typical shrub armed with short prickles on branches, which depends on epikarst water to cope with seasonal drought. The specific plant material monitored in this study is the ‘*dingtanensis*’ cultivar (*Zanthoxylum planispinum* var. *dingtanensis*). Although *Z. planispinum* is generally known as a deciduous species in temperate zones, the phenology of the ‘*dingtanensi*s’ cultivar is highly adaptive to local microclimates. According to local forestry guidelines (Forestry Bureau of Guizhou Province), in the subtropical karst dry-hot valleys—characterized by warm, virtually frost-free winters with extreme minimum temperatures consistently above 2 °C—this specific cultivar behaves as a semi-deciduous or broadleaf evergreen shrub. It maintains a photosynthetically active green canopy year-round without entering a distinct winter dormant period or experiencing massive leaf senescence. Therefore, the physiological and sap flow variations observed during the late autumn and winter months in this study are primarily driven by environmental water stress rather than intrinsic phenological dormancy. Long-term studies confirm its suitability to the climatic conditions of the study area, where it has been established as a major cash crop. Within the observation station, monitoring equipment for plant physiological ecology and environmental factors was installed in a *Z. planispinum* forest consisting of 7- to 8-year-old trees. In 2023, a natural drought event occurred in the study area, with precipitation plummeting from September and the annual precipitation reaching only 741.8 mm. The combined effects of reduced precipitation and karst topography make drought events particularly severe for local vegetation. Among the *Z. planispinum* trees instrumented with sap flow sensors, some have died due to the impact of natural drought events (hereinafter referred to as “dead *Z. planispinum*”), while others survived (hereinafter referred to as “surviving *Z. planispinum*”). Thus, under identical site conditions, the same species exhibited divergent outcomes in response to the natural drought event.

### 2.3. Research Method

#### 2.3.1. Environmental Factor Monitoring

Meteorological, plant physiological, and ecological data were monitored in real time using a Plant Physiological Ecology and Energy Balance System (model PE-PE07, Campbell Scientific, Inc., Logan, UT, USA), and the data were recorded every 30 min by the CR1000 data collector (Campbell Scientific, Inc., Logan, UT, USA). The meteorological data included precipitation, atmospheric pressure, air temperature, relative humidity (RH), and total radiation, among other variables. The vapor pressure deficit was calculated using the empirical formula described by Campbell and Norman [8]:

(1) $$VPD=0.61078e^{\frac{17.27T}{T_{a}+237.3}}(1-RH)$$

where VPD represents vapor pressure deficit, T represents atmospheric temperature, and RH represents relative humidity.

To quantitatively evaluate the intensity of the drought event and the physiological water stress experienced by the vegetation, the Standardized Evaporative Stress Ratio (SESR) was introduced. Unlike traditional meteorological drought indices (e.g., SPI) that rely solely on precipitation, SESR provides a robust indicator of environmental evaporative stress and has been widely used to assess the distribution and drivers of ecological and flash droughts [9,10]. This index directly reflects canopy stomatal regulation and plant moisture deficits.

In this study, the Evaporative Stress Ratio (ESR) is defined as the ratio of actual evapotranspiration (ET) to potential evapotranspiration (PET). Both ET and PET data were obtained from the MODIS Global Evapotranspiration Product (MOD16A2GF), which features a spatial resolution of 500 m × 500 m and a native 8-day temporal resolution [11,12]. The base SESR is first calculated by standardizing the ESR anomalies at the 8-day scale, expressed as follows:

(2) $${SESR}_{ijp}=\frac{{ESR}_{ijp}-\bar{{ESR}_{ijp}}}{\delta ESR}$$

where i and j represent the spatial grid coordinates, and p denotes the p-th octad (an 8-day averaging period, excluding leap days). The $\bar{{ESR}_{ijp}}$ and δESR represent the historical mean and standard deviation of the ESR for that specific grid point and octad, respectively. The temporal SESR data for the study area from August 2023 to March 2024 were extracted and analyzed. According to established drought classification criteria, an SESR value below −1.0 indicates moderate drought, a value below −1.5 denotes severe ecological drought, and a value below −2.0 represents extreme ecological drought.

#### 2.3.2. Sap Flow Monitoring in *Z. planispinum* Stems

**Figure 1:**
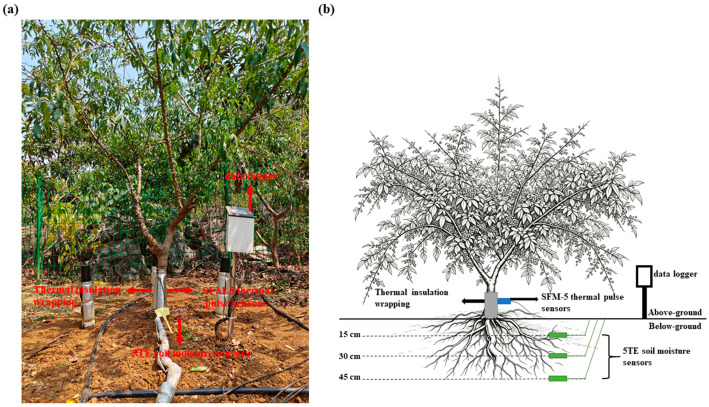
The in-situ monitoring system for *Zanthoxylum planispinum* var. *dingtanensis*. (**a**) Photograph of the field setup with major components directly labeled (e.g., SFM-5 thermal pulse sensors [UGT, Müncheberg, Germany], data logger enclosure, 5TE soil moisture sensors [METER Group, Inc., Pullman, WA, USA], and thermal insulation wrapping). (**b**) Schematic diagram illustrating the overall structural configuration and layout of the coupled sap flow and soil moisture monitoring system. (Disclosure: The line-drawing schematic in panel b was generated with the assistance of an AI tool based on the original field photograph.)

In this study, the five selected *Z. planispinum* trees had an average height of 2.02 m, an average basal diameter of 7.72 cm, and an average crown width of 2.5 m × 2.8 m (Table 1, Figure 1). The stand density of the *Z. planispinum* plantation was 2000 plants·hm^−2^. The selected individuals exhibited over 90% canopy integrity, minimal shading from neighboring plants, and no signs of pest or disease damage. Basal diameter was measured at 10 cm above ground level using a vernier caliper. Tree height was measured with a hypsometer, and crown width was recorded as the width in both the east–west and north–south directions.

Among these, two trees died during the observation period, leaving three survivors for data collection. Sap flow within the xylem was measured using stem flow sensors (SFM-5, UGT, Müncheberg, Germany). Each SFM-5 sensor comprises three probes (length: 30 mm, diameter: 1.6 mm) spaced 8 mm apart. The middle probe serves as a heating element, while the probes on both sides function as temperature measuring probes. Each probe contains both an outer and an inner thermistor, positioned 10 mm and 20 mm from the tip, respectively. The SFM-5 employs the thermal ratio method—an improved variant of the thermal pulse technique—to measure sap flow. In this method, a short heat pulse is applied to the stem, and temperature probes placed equidistantly above and below the heater measure the upward and downward heat movement along the stem axis. During installation, sapwood thickness was first determined. The phloem was then carefully removed to expose the xylem. A positioning template was used to align the probe holes, which were drilled into the xylem to the mid-sapwood depth using a micro-drill before inserting the probes. To minimize solar radiation effects, all probes were installed on the north side of each stem. After installation, drilled holes were sealed with silicone to prevent moisture intrusion or resin leakage. And the exposed probe section was wrapped with reflective foil to prevent thermal interference from environmental temperature. To ensure data accuracy and stability, measurements were taken every 10 s, and 30 min averages were recorded using a data collector (CR1000, Campbell Scientific, Inc., Logan, UT, USA). The thermal pulse velocity (cm·h^−1^) was calculated according to the following formula [13]:

(3) $$V_{h}=k/x\times \ln\left(v_{1}/v_{2}\right)\times 3600$$

where $V_{h}$ represents the thermal pulse velocity. k represents the thermal diffusivity of fresh wood, with a standard value of 2.5 × 10^−3^ cm^−2^·s^−1^ [13]. x represents the distance (in cm) between the heating probe and any temperature measuring probe, with the spacing between SFM-5 sensor probes being 0.6 cm. v_1_ and v_2_ represent the differences between the initial temperature (°C) of the two heating probes and the temperature measured after the thermal pulse.

The thermal pulse velocity technique is highly sensitive to errors in probe spacing, and inaccurate probe spacing will lead to overestimation or underestimation of sap flow measurements. Therefore, the following equation is used for correction:

(4) $$V_{h}=\left[4kt\;\ln\left(v_{1}/v_{2}\right)-\left(x_{2}^{2}\right)+\left(x_{1}^{2}\right)\right]/2t\left(x_{1}-x_{2}\right)\times 3600$$

where $V_{h}$ represents the thermal pulse velocity. t represents the measurement time. Assuming that x_1_ has the correct spacing at 0.6 cm, x_2_ represents the probe with incorrect spacing. By correcting a small portion of the data samples and then comparing the corrected values with the uncorrected values, a linear relationship is fitted to correct the remaining data.

The installation of sensors inevitably causes significant wounding to the xylem. Without correction, it will lead to errors in measurement results [14,15]. Therefore, the model developed by Burgess et al. is utilized for Wound Correction [15].

(5) $$V_{c}=bV_{h}+cV_{h}^{2}+dV_{h}^{3}$$

where $V_{c}$ represents the corrected thermal pulse velocity, and b, c, and d are the correction coefficients.

Only a portion of the xylem tissue contains flowing sap [15]. The thermal pulse probe effectively measures the weighted average of sap flow velocities, both high and low [13]. By measuring the components of sap and wood in the xylem, and considering their different densities and specific heat capacities, the sap flow velocity can be determined on an area basis. The sap flow velocity was calculated using the formula refined by Barrett et al. [14].

(6) $$V_{s}=\left[v_{c}\rho_{b}\left(c_{w}+m_{c}c_{s}\right)\right]/\rho_{s}c_{s}$$

where $V_{s}$ represents the sap flow velocity. $\rho_{b}$ represents the basic density of wood (dry weight/green volume), $c_{w}$ and $c_{s}$ are the specific heat capacities of the wood matrix (1200 J·kg^−1^·°C^−1^ at 20 °C [16]) and sap flow (water, 4182 J·kg^−1^·°C^−1^ at 20 °C [17]), $m_{c}$ is the moisture content of wood, and *ρ_s_* is the density of water.

The sap flow rate can monitor the real-time status of water transport in trees. The sap flow volume (Q) of an entire tree is calculated using the following formula:

(7) $$Q=V_{s}\times A_{s}$$

where $V_{s}$ is the sap flow velocity and $A_{S}$ is the cross-sectional area of the hydraulically active sapwood.

Prior to the calculations using Equations (3)–(7), the raw sap flow data under RH underwent a rigorous validation and filtering procedure. First, abnormal spikes and unrealistic negative values caused by power fluctuations or sensor malfunctions were screened and removed. Missing data gaps shorter than 2 h were filled using linear interpolation, while larger gaps were excluded from the analysis. Second, to eliminate potential baseline drift, a zero-flow calibration (baseline subtraction method) was performed. The zero-sap-flow state was determined during nights with near-zero transpirational demand, specifically during late-night periods (00:00–04:00) with continuous rainfall, when VPD was <0.1 kPa and RH exceeded 95%. This established baseline was subsequently subtracted from the raw thermal pulse velocities to ensure the true zero point was accurately represented across the entire observation period.

#### 2.3.3. Soil Moisture Monitoring

Within a 50 cm radius of the *Z. planispinum* tree, soil moisture sensors (5TE, METER Group, Inc., Pullman, WA, USA) were installed at depths of 15 cm, 30 cm, and 45 cm to monitor soil moisture. Data was recorded every 30 min by a ZL6 data collector (METER Group, Inc., Pullman, WA, USA).

#### 2.3.4. Measurement of Xylem Embolism Vulnerability Curve

Three well-grown sample trees were selected from the sample plot, and from each tree, six sun-exposed branches growing in different directions were collected. Upon excision, the branches were immediately immersed in pure water, enclosed in black plastic bags, and transported in a cooler to the laboratory. Before measurement, 5–7 cm was trimmed off both ends of the branches underwater. The final measured branch length is 12–15 cm. Prior to the construction of the vulnerability curves, the maximum vessel length (MVL) of *Z. planispinum* branches was determined using the standard air infiltration method. Branch segments (~50 cm) were pressurized with air at 50 kPa from the proximal end, while the distal end was submerged in water and successively cut back by 1 cm increments until a steady stream of bubbles appeared. Preliminary measurements indicated that the MVL of the sampled *Z. planispinum* trees was approximately 9.3 ± 1.2 cm. Therefore, a sample length of 12–15 cm was strictly chosen to be longer than the MVL, effectively avoiding the open-vessel artifact during hydraulic conductivity measurements. Furthermore, the vulnerability curves were constructed using the cumulative progressive pressurization method (independent branches were not used for each pressure step), with a total of 6 branch samples. The xylem embolism and hydraulic conductivity measurement system (model XYLEM-Plus, Xylem, Paris, France) was used to remove xylem embolism of *Z. planispinum* and measure its xylem hydraulic conductivity. First, the excised branches were flushed with high-pressure water (100 kPa) for 10 min to remove potential xylem embolisms, prior to measuring the maximum xylem hydraulic conductivity ($K_{smax}$) under low-pressure water. A portable plant water potential pressure chamber (model 1000, PMS Instrument Company, Albany, OR, USA) was used to apply pressure to the branches, inducing embolism in the xylem at different pressure values. Then, the branches with established maximum hydraulic conductivity ($K_{smax}$) were transferred to a pressure chamber, pressurized to 50 kPa, and held at this pressure for 10 min to induce embolism. The resulting xylem hydraulic conductivity ($K_{s}$) was immediately measured under low-pressure flow using a XYLEM-Plus system. Following the measurement, the induced embolism was cleared by performing two high-pressure flush cycles. Then, the pressure was incrementally increased in 50 kPa steps up to 450 kPa, with the embolism induction and hydraulic conductivity measurement procedure repeated at each pressure level. The formula for calculating the percent loss of conductivity (PLC) of plant hydraulic conductivity is as follows:

(8) $$PLC=100\times (1-\frac{K_{s}}{K_{smax}})$$

Among them, $K_{s}$ refers to the hydraulic conductivity of the xylem of *Z. planispinum* after inducing embolism under different pressures, and $K_{smax}$ refers to the maximum hydraulic conductivity of the xylem of *Z. planispinum*.

#### 2.3.5. Estimation of Hydraulic Safety Margin

During the extreme drought period (November 2023), the minimum field branch water potential ($\psi_{min}$) of *Z. planispinum* was measured at midday (12:00–14:00) on clear days using a portable pressure chamber (Model 1000, PMS Instrument Company, Albany, OR, USA). To accurately represent the xylem tension, terminal shoots were enclosed in opaque, impermeable bags for at least 30 min prior to excision to ensure equilibration between leaf and branch xylem water potentials.

The hydraulic safety margin (HSM) quantifies the safety buffer between natural drought stress and hydraulic failure, serving as a key predictive indicator of species mortality [18]. The correct calculation formula is:

(9) $$HSM=\psi_{min}{\;-\;P}_{50}$$

where $P_{50}$ represents the xylem water potential at which 50% of hydraulic conductivity is lost, derived from the vulnerability curve. A negative HSM indicates that the plant’s field water tension has exceeded its $P_{50}$ threshold, signifying severe embolism and a high risk of lethal hydraulic failure [19].

#### 2.3.6. Data Analysis

Field monitoring data were processed using Excel 2021. Pearson correlation analysis was conducted to examine the correlation between sap flow and environmental factors. A Random Forest model was employed in R (version 3.6.1) to establish a predictive model between environmental factors and sap flow, aiming to identify key drivers influencing the mortality and survival of *Z. planispinum*. The xylem vulnerability curve was fitted and visualized using Origin 2021. Additionally, an Independent-Samples t-test was used to evaluate the statistical significance of differences in sap flow rates between the surviving and dead *Z. planispinum* individuals.

## 3. Results and Analysis

### 3.1. Meteorological Dynamic Characteristics

**Figure 2:**
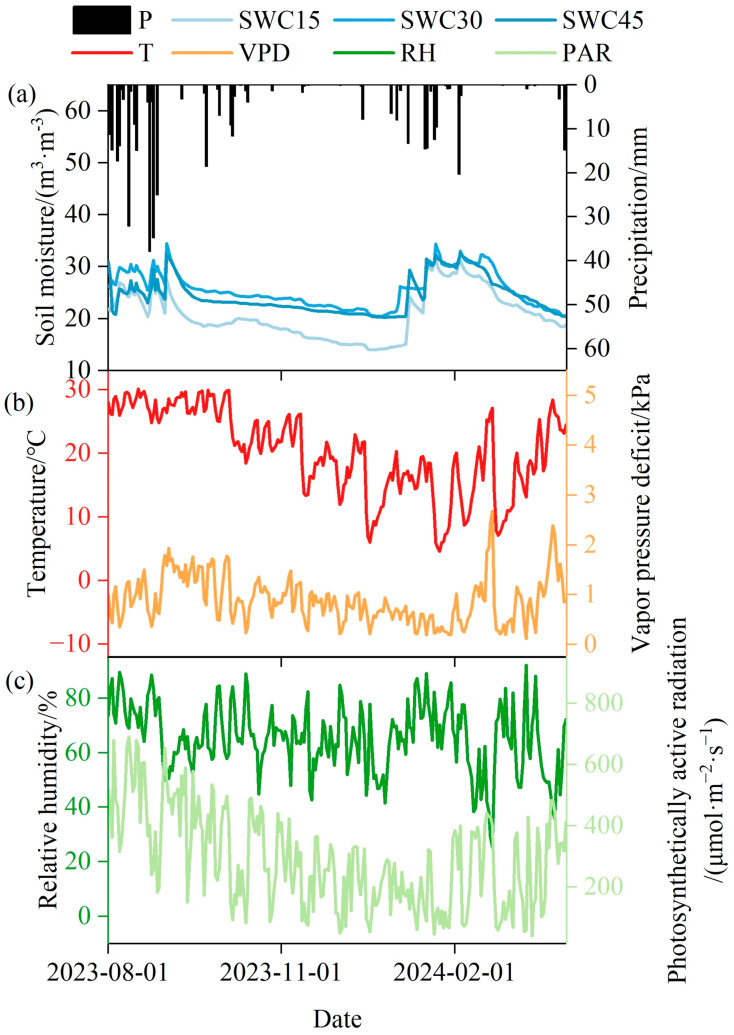
Dynamics of environmental factors in the study area from August 2023 to March 2024. (**a**) Daily cumulative precipitation (P) and daily mean soil moisture content at 15, 30, and 45 cm depths (SWC15, SWC30, SWC45). (**b**) Daily mean air temperature (T) and daily mean vapor pressure deficit (VPD). (**c**) Daily mean relative humidity (RH) and daily mean photosynthetically active radiation (PAR).

Figure 2 illustrates the dynamic variations in temperature, RH, photosynthetically active radiation (PAR), VPD, and precipitation in the study area from August 2023 to March 2024. Temperature peaked in September and subsequently declined to its lowest point in January. Both RH and PAR exhibited a gradual decreasing trend throughout the observation period. VPD showed an initial rise, followed by a decline, and then a gradual increase thereafter. From August to September, the combined effects of declining RH and rising temperatures contributed to an upward trend in VPD. Precipitation in the study area was primarily concentrated between May and October. However, in 2023, precipitation decreased abruptly in September and continued to decline in the following months, reaching a minimum of only 2.1 mm in November.

Soil moisture dynamics in *Z. planispinum* fields are generally consistent with precipitation. The highest precipitation in August corresponded with the peak soil moisture. Subsequently, as precipitation decreases, soil moisture content also decreases. The lowest soil moisture was recorded in December, which did not coincide with the period of *Z. planispinum* mortality observed in November. Furthermore, under high precipitation conditions, shallow soil moisture was lower than that in middle and deep soil layers; conversely, during low precipitation periods, shallow soil moisture exceeded that in middle and deep layers.

Instead of visual representation, the trajectory of the SESR was quantitatively tracked to confirm the severity of this natural drought event. During the initial phase in August 2023, the SESR was already at −1.50, indicating an early onset of severe evaporative stress. Driven by the abrupt precipitation deficit and persistent intense thermal radiation from the karst rocky environment, the drought rapidly intensified. Crucially, in November—the exact temporal window when the catastrophic sap flow cessation and mortality of *Z. planispinum* were observed—the SESR plummeted to an extreme nadir of −2.36. According to the standardized drought classification matrix, values below −2.0 unequivocally confirm a state of extreme ecological drought (Extreme drought). This period of lethal physiological water stress persisted through December 2023 (SESR = −2.19) before showing initial signs of alleviation in early 2024.

### 3.2. Sap Flow Dynamics Preceding and Following Z. planispinum Mortality

**Figure 3:**
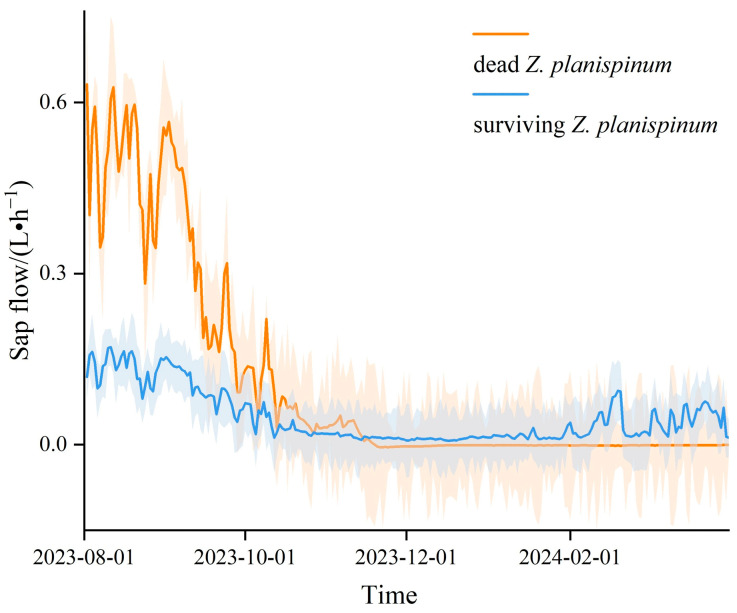
Sap flow dynamics of the dead (*n* = 2) and surviving (*n* = 3) *Zanthoxylum planispinum* var. *dingtanensis* individuals during the observation period. The solid curves represent the daily mean sap flow volume for each respective group. The shaded bands denote the standard error (SE) of the mean, illustrating the biological variability among the monitored individuals.

From the comparison of sap flow dynamics between dead and alive *Z. planispinum* (Figure 3), it can be observed that the total sap flow showed a continuous downward trend before the death of the *Z. planispinum*. However, at the same sampling site, the sap flow of dead individuals was not only higher in magnitude but also characterized by a wider fluctuation range and multiple distinct peaks, rendering their sap flow pattern highly unstable. The maximum sap flow of the dead *Z. planispinum* reached 0.63 L/h, whereas that of the surviving *Z. planispinum* was merely 0.17 L/h, indicating a nearly 3.7-fold significant difference between the two (*p* < 0.01). As detailed in Table 1, the sapwood areas across all monitored individuals were highly consistent (ranging minimally from 44.18 to 50.27 cm^2^). Therefore, this massive disparity in total sap flow volume was not a morphological artifact caused by larger tree sizes, but rather strictly driven by higher intrinsic sap flux density. This confirms that the deceased individuals maintained a highly acquisitive physiological transpiration strategy under drought stress. In November, pronounced fluctuations in sap flow were detected across all monitored *Z. planispinum* individuals. After 11 November, the sap flow of dead *Z. planispinum* declined progressively, and by 19 November, sap flow ceased entirely, marking the complete mortality of these individuals. In response to drought stress, the sap flow of surviving *Z. planispinum* also displayed a sustained decreasing trend, maintaining a low-flow state throughout the dry season. It was not until January that the sap flow of surviving *Z. planispinum* initiated a recovery and upward trajectory.

### 3.3. The Driving Effect of Environmental Factors on the Sap Flow of Z. planispinum

**Figure 4:**
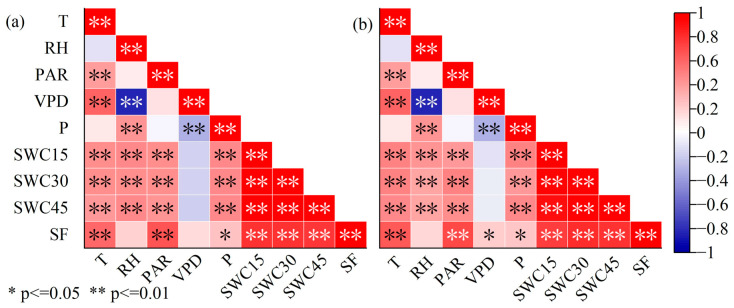
Pearson correlation analysis between environmental factors and sap flow for (**a**) dead *Zanthoxylum planispinum* var. *dingtanensis* (*n* = 2) and (**b**) surviving *Zanthoxylum planispinum* var. *dingtanensis* (*n* = 3). The color scale represents the Pearson correlation coefficient (r). Red gradients indicate positive correlations, while blue gradients indicate negative correlations. Significance levels are denoted by asterisks: * *p* ≤ 0.05 and ** *p* ≤ 0.01. Abbreviations: T, temperature; RH, relative humidity; PAR, photosynthetically active radiation; VPD, vapor pressure deficit; P, precipitation; SWC15, SWC30, and SWC45, soil moisture at 15, 30, and 45 cm depths, respectively; SF, sap flow.

According to the results of Pearson correlation analysis, the sap flow of dead *Z. planispinum* individuals (Figure 4a) exhibited a highly significant positive correlation with temperature, photosynthetically active radiation, and soil moisture across all three layers, and a significant positive correlation with precipitation. In contrast, no significant correlation was detected between its sap flow and either RH or VPD. For surviving *Z. planispinum* individuals (Figure 4b), sap flow demonstrated a highly significant positive correlation with temperature, PAR, soil moisture across all three layers; additionally, it showed a significant positive correlation with VPD and precipitation, while no significant correlation was observed with RH.

**Figure 5:**
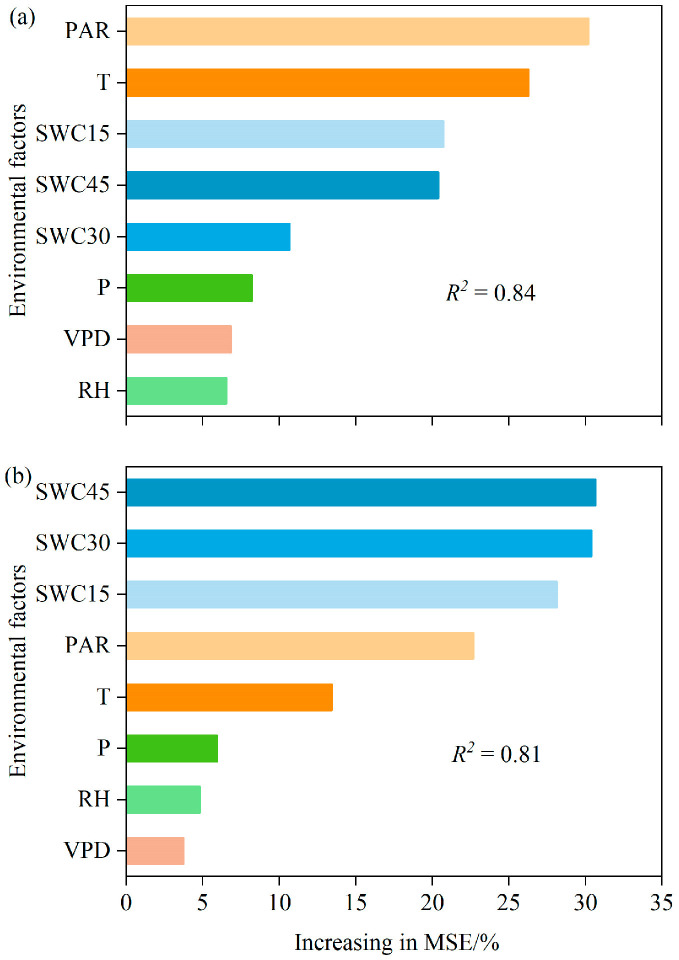
The relative importance of environmental factors driving the sap flow of (**a**) dead *Zanthoxylum planispinum* var. *dingtanensis* (*n* = 2) and (**b**) surviving *Zanthoxylum planispinum* var. *dingtanensis* (*n* = 3), determined via Random Forest model analysis. The x-axis represents the percentage increase in Mean Squared Error (MSE), where higher values indicate greater variable importance in the model. The reported R^2^ values represent the proportion of variance in sap flow explained by the respective models. (Abbreviations match those in Figure 4).

The results of random forest model analysis indicated that environmental factors exhibited significant differences in driving the sap flow dynamics of dead and surviving *Z. planispinum* individuals (Figure 5). For dead *Z. planispinum*, the relative importance of environmental factors was ranked in descending order as follows: PAR > air temperature > 0–15 cm soil moisture >30–45 cm soil moisture >15–30 cm soil moisture > precipitation > VPD > RH. In contrast, for surviving *Z. planispinum*, the ranking of environmental factors by relative importance (descending) was: 30–45 cm soil moisture >15–30 cm soil moisture > 0–15 cm soil moisture > PAR > air temperature > precipitation > RH > VPD. These results indicate that sap flow in dead *Z. planispinum* was mainly driven by meteorological factors, whereas soil moisture played the dominant role in regulating sap flow in surviving *Z. planispinum*.

### 3.4. Analysis of the Fragility Curve of Z. planispinum

**Figure 6:**
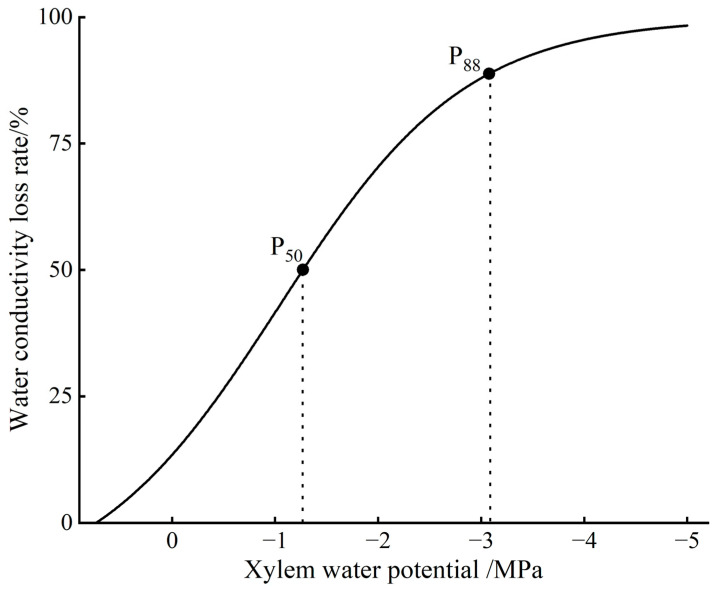
Xylem vulnerability curve of *Zanthoxylum planispinum* var. *dingtanensis* (*n* = 6 independent branches). The curve represents the percentage loss of hydraulic conductivity (PLC) in response to progressively declining xylem water potential. $P_{50}$ (−2.2 MPa) indicates the critical water potential inducing a 50% loss of conductivity, representing the threshold of substantial xylem embolism. $P_{88}$ (−4.1 MPa) indicates the threshold for 88% loss of conductivity, widely recognized as the lethal limit for angiosperms.

As illustrated in Figure 6, with the continuous decline of xylem water potential, the vulnerability curve of *Z. planispinum* exhibited a characteristic sigmoidal shape, indicating a non-linear response of the hydraulic system to progressive water stress. The critical xylem water potential inducing 50% loss of hydraulic conductivity ($P_{50}$, the threshold of substantial embolism) was determined to be −2.2 MPa. As the tension intensified, the curve steepened rapidly, with the lethal threshold inducing 88% loss of conductivity ($P_{88}$) reaching −4.1 MPa. Furthermore, during the peak of the severe drought event (November 2023), in situ measurements revealed that the minimum field branch water potential ($\psi_{min}$) of the stressed *Z. planispinum* dropped significantly to −3.0 MPa. By integrating the field water potential with the inherent vulnerability trait, the HSM (HSM = $\psi_{min}$ − $P_{50}$) was calculated as −0.8 MPa. This highly negative HSM indicates that the actual xylem tension experienced by the plants in the field severely breached their 50% embolism resistance threshold, resulting in a massive and potentially irreversible loss of xylem hydraulic conductivity.

## 4. Discussion

### 4.1. Differential Responses of Sap Flow in Z. planispinum to Environmental Factors Under Drought Stress

The divergent response of sap flow in *Z. planispinum* to environmental factors ultimately leads to contrasting outcomes. As soil moisture declines, surviving *Z. planispinum* exhibits a prompt response to water supply fluctuations, with soil moisture emerging as the primary limiting factor regulating sap flow dynamics. In contrast, meteorological factors remain the dominant drivers of *Z. planispinum* mortality during this period. However, sap flow in dead *Z. planispinum* remains significantly influenced by meteorological factors during this period. Previous studies indicate that plant sap flow is co-regulated by meteorological factors and soil moisture [20]. Under sufficient soil moisture conditions, sap flow is primarily driven by meteorological variables such as air temperature, PAR, and VPD [21]. Elevated air temperature, PAR, and VPD typically promote stomatal opening, enhance transpiration efficiency, and facilitate photosynthesis and plant growth metabolism [22,23]. However, as soil moisture decreases, the correlation between sap flow and meteorological factors weakens, and soil moisture becomes the key limiting factor for sap flow dynamics [20]. Soil moisture serves as a critical water source for plant growth, and its status and distribution directly influence plant performance. In the study area, despite a gradual reduction in precipitation, PAR and air temperature remained persistently high. Under water-limited conditions, sustained high levels of these meteorological factors exacerbate drought stress by increasing plant transpiration rates, thereby restricting plant growth and recovery and exerting adverse effects on plant vitality [24]. Without timely water supplementation, plants face an elevated risk of “hydraulic failure” [25], which can lead to mortality in severe cases. Notably, the annual precipitation in the study area in 2023 was only 741.8 mm—nearly 33% lower than the long-term annual average. Precipitation in September and October plummeted to a mere 61.4 mm, with a minimum of 2.1 mm recorded in November. The continuous precipitation deficit hindered timely soil moisture replenishment, resulting in insufficient soil water availability for *Z. planispinum*. This posed a severe threat to *Z. planispinum* survival and ultimately led to mortality in some individuals. Given the aforementioned drought stress and associated hydraulic risks, estimating the HSM is therefore particularly crucial for sustaining the normal growth and survival of *Z. planispinum*.

### 4.2. The Impact of Drought on the Dynamics of Sap Flow and Hydraulic Safety Margin in Z. planispinum

The inherent hydraulic traits of *Z. planispinum* provide the fundamental physiological context for elucidating its drought response and subsequent mortality. Based on our vulnerability curve measurements, the species exhibited a $P_{50}$ of −2.2 MPa. While this value indicates a moderate baseline drought tolerance adapted to the typical karst dry-hot valley environment [19,26], the remarkably narrow margin (1.9 MPa) between the onset of major cavitation ($P_{50}$) and the lethal threshold ($P_{88}$ = −4.1 MPa) reflects a characteristically steep vulnerability curve. According to Martin-StPaul et al. [27], a steep vulnerability curve implies a highly limited physiological buffer. Once the initial embolism threshold is crossed, the xylem vessel network is extraordinarily susceptible to rapid, runaway cavitation due to the exponential spread of air-seeding events across adjacent pit membranes. During the severe autumn drought of 2023, the extreme atmospheric evaporative demand (quantified by a severely negative SESR of −2.36) subjected the trees to unprecedented transpirational pull. Under this intense environmental stress, our field measurements confirmed that the minimum branch water potential ($\psi_{min}$) of the stressed individuals plummeted to −3.0 MPa. By integrating the field water potential with the inherent vulnerability trait, the true HSM was calculated as −0.8 MPa. According to classic drought mortality frameworks [18,28], a negative HSM is a definitive hallmark of imminent hydraulic failure. This highly negative margin demonstrates that the actual xylem tension (−3.0 MPa) severely breached the $P_{50}$ defense line (−2.2 MPa), introducing massive and irreversible air embolisms into the xylem conduits. The contrasting sap flow dynamics observed between the surviving and deceased individuals prior to mortality can be mechanistically explained by divergent functional trait strategies across the isohydric-anisohydric continuum [29,30]. The surviving trees likely adopted a “conservative” (isohydric-like) water-use strategy, characterized by high stomatal sensitivity to rising VPD and declining soil moisture. By downregulating sap flow early during drought onset, these survivors conserved intrinsic plant water reserves, thereby maintaining their xylem tension safely above the critical $P_{50}$ threshold. Conversely, the deceased trees exhibited an “acquisitive” or anisohydric-like profligate water-use strategy. Although direct leaf-level stomatal conductance was not measured, the whole-tree sap flow dynamics act as a robust proxy for canopy stomatal sensitivity. The continuous driving of sap flow by meteorological factors—rather than soil moisture—indicates extremely low stomatal sensitivity in these deceased individuals. In a bid to prioritize carbon assimilation and maintain metabolic functions, these individuals failed to promptly close their stomata, resulting in anomalously high transpiration rates despite the severe soil water deficit [31,32]. Under the extreme atmospheric evaporative demand of the karst environment, this acquisitive stomatal behavior became profoundly maladaptive, accelerating the depletion of internal water storage and directly driving the $\psi_{min}$ to the lethal −3.0 MPa limit. Crucially, the paradoxical sap flow pattern—maintaining highly fluctuating sap flow even after severe soil moisture depletion—points to a deeper physiological failure that contradicts typical plant water conservation strategies. While the initial high transpiration was driven by an acquisitive strategy, the subsequent inability to downregulate sap flow likely indicates severe stomatal dysfunction. As xylem tension plummeted towards the lethal threshold, guard cells may have experienced extreme loss of turgor or mechanical damage, resulting in a physiological “lock-up” state that prevented stomatal closure [33]. Alternatively, once catastrophic partial cavitation occurred, the observed fluctuations in sap flow might no longer represent active physiological transpiration. Instead, they could reflect passive water movement or uncontrolled cuticular water loss driven by the extremely high VPD, physically emptying the remaining water from the stem storage tissues [34]. Moreover, this above-ground physiological dysregulation must be contextualized within the high spatial heterogeneity of karst subterranean environments, where undetected root system pathogens or highly restricted rooting depth could have further exacerbated their water stress. As highlighted by recent ecohydrological studies in karst rocky desertification regions, plant survival heavily depends on root access to deep epikarst water or bedrock fissures [35,36]. It is highly plausible that the surviving *Z. planispinum* individuals had developed deeper root systems capable of tapping into stable subsurface fissure water, effectively mitigating the shallow soil drought. In contrast, the deceased trees might have possessed shallower root systems restricted to the 0–45 cm soil layer. Once this shallow soil moisture was completely depleted under the high SESR, their acquisitive stomatal strategy left them with no alternative water source to buffer the escalating xylem tension. Ultimately, this relentless transpirational pull, coupled with the lack of adequate soil water replenishment, irrevocably severed the soil–plant–atmosphere continuum (SPAC). The uncontrolled runaway cavitation completely disrupted the hydraulic transport architecture, culminating in the sudden, catastrophic cessation of sap flow and the ultimate mortality of the *Z. planispinum* individuals [37]. These findings suggest that under future climate change scenarios, which predict an increased frequency of extreme flash droughts (high SESR), species with narrow HSMs and acquisitive water-use strategies face severe mortality risks in shallow karst soils.

### 4.3. Implications for Future Z. planispinum Plantation

Sap flow of surviving *Z. planispinum* is predominantly regulated by soil water, while sap flow of dead individuals is persistently driven by meteorological factors, indicating that soil water supply capacity constitutes the core threshold for the drought survival of *Z. planispinum*. The study area is located in the karst plateau canyon. Although annual precipitation is sufficient, high air temperature causes most infiltrated rainfall to be depleted within two weeks via evaporation, transpiration, or deep percolation [38]. Soil water evaporates and dissipates rapidly, resulting in severe water shortages for most crops [7]. After drought onset, persistently high and sharply fluctuating sap flow in *Z. planispinum* serves as an early warning signal of impending hydraulic failure and plant mortality. In cultivation and production, the stem sap flow rate can be adopted as an irrigation trigger threshold. When stem sap flow fluctuates drastically and remains long-term within the range of 0.17–0.63 L·h^−1^, artificial water replenishment should be implemented immediately. Random Forest model analysis in this study indicates that soil water content at the 30–45 cm layer is the primary driver of sap flow in surviving *Z. planispinum* individuals. Accordingly, subsurface infiltration irrigation is recommended, with drip pipes directly buried at a soil depth of 30–45 cm to preferentially replenish soil water in this layer. Meanwhile, moss planting on the soil surface can mitigate evaporation from the 0–30 cm soil layer and rapidly alleviate the imbalance between water supply and demand. For newly planted *Z. planispinum* in the future, gentle slope sites with soil thickness ≥ 30 cm and strong water retention capacity should be prioritized. Drip irrigation pipes can be pre-arranged in the 30–45 cm soil layer. Additionally, appropriate planting density, inter-row grass cover, and seasonal pruning practices are suggested to reduce belowground water competition among individuals and decrease canopy transpiration load. These measures can prevent large-scale yield reduction and mortality of *Z. planispinum* caused by long-term hydraulic imbalance.

## 5. Conclusions

Compared to surviving individuals, the dead *Z. planispinum* exhibited highly fluctuating and anomalously high sap flow rates prior to mortality. This suggests that these plants maintained an acquisitive water-use strategy, sustaining significant transpiration despite severe drought conditions. During this period, the sap flow of the dead *Z. planispinum* failed to downregulate in response to soil moisture depletion, remaining primarily driven by atmospheric demand. Coupled with the severe scarcity of soil water, this continuous transpirational pull could not be replenished, driving internal water potentials to critically low levels. Furthermore, the xylem vulnerability curve analysis revealed a $P_{50}$ of −2.2 MPa. Combined with field measurements, the resulting negative HSM (HSM = −0.8 MPa) strongly indicates that *Z. planispinum* experienced severe runaway cavitation, firmly implicating catastrophic hydraulic failure as the primary mechanism for the observed mortality. While these field observations provide crucial high-resolution insights into drought-induced mortality, they also highlight the extreme vulnerability of species with narrow HSMs. Therefore, following the onset of natural flash droughts, it is highly recommended to continuously monitor plant sap flow and environmental water conditions. For species exhibiting maladaptive acquisitive water-use patterns under drought stress, timely artificial interventions (e.g., supplemental irrigation) are essential to prevent irreversible hydraulic failure. These insights hold significant practical value for the restoration, management, and protection of karst plantation ecosystems under increasingly frequent climate-change-induced droughts.

## 6. Limitations and Future Directions

While this study provides rare, high-resolution, continuous sap flow observations capturing the exact physiological progression of drought-induced mortality in situ, we explicitly acknowledge the severe statistical constraints imposed by our limited sample size (*n* = 5, with two deceased and three surviving individuals). Installing and maintaining delicate ecophysiological monitoring equipment in harsh, highly heterogeneous karst terrain precludes large-scale population sampling. Furthermore, capturing tree mortality “in the act” using pre-installed sap flow sensors relies heavily on opportunistic extreme weather events. Consequently, the distinct ecohydrological thresholds and divergent survival strategies observed in this study should be interpreted as an illustrative, high-resolution case study rather than definitive, species-wide parameters. Specifically, given that the paradoxical sap flow pattern—maintaining high and fluctuating transpiration despite severe drought—was observed in only two eventually deceased individuals, the associated mechanistic hypotheses regarding stomatal dysfunction must be considered preliminary. Future studies with larger sample sizes, concurrent measurements of leaf-level minimum conductance, and root health assessments are urgently required for robust verification. Furthermore, our monitoring focused primarily on whole-tree hydraulic dynamics without synchronous measurements of leaf-level functional traits, such as Specific Leaf Area (SLA) or direct stomatal conductance. The absence of these morpho-physiological traits limits a more comprehensive understanding of the carbon-water trade-off mechanisms prior to mortality. Extrapolating these specific thresholds to the entire *Z. planispinum* population or partitioning environmental drivers with high statistical certainty requires caution.

Additionally, we must explicitly acknowledge a structural uncertainty regarding our soil moisture monitoring. Due to the extreme shallowness of karst surface soils, our sensors were structurally limited to a maximum depth of 45 cm, immediately above the carbonate bedrock interface. Therefore, the identification of the 30–45 cm layer as the primary soil-water driver for surviving trees merely reflects the deepest continuous soil profile monitored. It is highly probable that surviving individuals also utilized specialized dimorphic root systems penetrating deep bedrock fissures to access subsurface epikarst water—a critical survival dynamic not captured by our shallow soil sensors. Extrapolating these specific thresholds to the entire *Z. planispinum* population or partitioning environmental drivers with high statistical certainty requires caution.

To overcome these limitations, future research should integrate our high-temporal-resolution physiological data with larger-scale, multi-site surveys. Analytically, employing advanced machine learning frameworks, such as XGBoost coupled with SHapley Additive exPlanations (SHAP), will be crucial to unravel complex non-linear tipping points and synergistic interactions among environmental drivers across diverse karst landscapes. Finally, to address the spatial limitations of plot-level studies and the high heterogeneity of karst rocky desertification areas, future efforts must integrate high-resolution UAV-based remote sensing. Specifically, monitoring the Kinematic Normalized Difference Vegetation Index (KNDVI) during post-drought recovery periods will be essential to bridge the gap between tree-level hydraulic failure and broader canopy-level ecohydrological resilience. Moreover, applying stable isotope tracing techniques in future studies will be vital to accurately partition the contributions of shallow soil moisture versus deep epikarst water.

## Figures and Tables

**Table 1:** Basic information of *Zanthoxylum planispinum* var. *dingtanensis* trees.

No.	Tree Height (m)	Basal Diameter (cm)	Crown Width (m)	Sapwood Area (cm^2^)
1	1.8	7.6	2.2 × 3.1	45.36
2	2	7.7	2.4 × 2.9	46.57
3	2.1	7.5	2.9 × 2.8	44.18
4	2.2	7.8	2.3 × 3	47.78
5	2	8	3.1 × 2.6	50.27

## Data Availability

The datasets presented in this article are not readily available because of confidentiality agreements with participants and institutional data protection policies. Requests to access the datasets should be directed to the corresponding author.

## References

[1] Huang J., Yu H., Guan X., Wang G., Guo R. (2016). Accelerated dryland expansion under climate change. Nat. Clim. Change.

[2] Novick K.A., Ficklin D.L., Stoy P.C., Williams C.A., Bohrer G., Oishi A.C., Papuga S.A., Blanken P.D., Noormets A., Sulman B.N. (2016). The increasing importance of atmospheric demand for ecosystem water and carbon fluxes. Nat. Clim. Change.

[3] Jiang Z., Lian Y., Qin X. (2014). Rocky desertification in Southwest China: Impacts, causes, and restoration. Earth-Sci. Rev..

[4] Sperry J.S., Love D.M. (2015). What plant hydraulics can tell us about responses to climate-change droughts. New Phytol..

[5] Hasselquist N.J., Allen M.F., Santiago L.S. (2010). Water relations and hydraulic architecture of woody plants in a karst ecosystem. Tree Physiol..

[6] Wang C., Fu B., Zhang L., Xu Z. (2019). Soil moisture–plant interactions: An ecohydrological review. J. Soils Sediments.

[7] Liu Z.Q., Li K.P., Xiong K.N. (2021). Effects of *Z. planispinum* planting on soil hydraulic properties and soil moisture in a karst area. Agric. Water Manag..

[8] Campbell G.S., Norman J.M. (2000). An Introduction to Environmental Biophysics.

[9] Christian J.I., Basara J.B., Hunt E.D., Otkin J.A., Furtado J.C., Mishra V., Randall R.M. (2021). Global distribution, trends, and drivers of flash drought occurrence. Nat. Commun..

[10] Edris S.G., Basara J.B., Christian J.I., Hunt E.D., Otkin J.A., Salesky S.T., Illston B.G. (2023). Analysis of the critical components of flash drought using the standardized evaporative stress ratio. Agric. For. Meteorol..

[11] Christian J.I., Basara J.B., Otkin J.A., Hunt E.D., Wakefield R.A., Flanagan P.X., Xiao X. (2019). A methodology for flash drought identification: Application of flash drought frequency across the United States. J. Hydrometeorol..

[12] Yang C.H., Li D.K., Zhou X.T., Zhang C.F., Zheng K., Wang Q. (2025). Rapid identification and evolution analysis of flash droughts in the eastern coast of China. J. Hydrol..

[13] Marshall D.C. (1958). Measurement of sap flow in conifers by heat transport. Plant Physiol..

[14] Barrett D.J., Hatton T.J., Ash J.E., Ball M.C. (1995). Evaluation of the heat pulse velocity technique for measurement of sap flow in rainforest and eucalypt forest species of south-eastern Australia. Plant Cell Environ..

[15] Burgess S.S.O., Adams M.A., Turner N.C., Beverly C.R., Ong C.K., Khan A.A.H., Bleby T.M. (2001). An improved heat pulse method to measure low and reverse rates of sap flow in woody plants. Tree Physiol..

[16] Becker P., Edwards W.R.N. (1999). Corrected heat capacity of wood for sap flow calculations. Tree Physiol..

[17] Lide D.R. (1992). CRC Handbook of Chemistry and Physics.

[18] Anderegg W.R.L., Klein T., Bartlett M., Sack L., Adam F.A.P., Brendan C., Steven J. (2016). Meta-analysis reveals that hydraulic traits explain cross-species patterns of drought-induced tree mortality across the globe. Proc. Natl. Acad. Sci. USA.

[19] Choat B., Jansen S., Brodribb T.J., Cochard H., Delzon S., Bhaskar R., Bucci S.J., Feild T.S., Gleason S.M., Hacke U.G. (2012). Global convergence in the vulnerability of forests to drought. Nature.

[20] Zhao X., Fan J. (2024). Response of tree sap flow rate to soil water and atmospheric environment, and adaptability to drought in the Loess Plateau region of China. For. Ecol. Manag..

[21] Zeng X., Xu X., Yi R., Zhong F., Zhang Y. (2021). Sap flow and plant water sources for typical vegetation in a subtropical humid karst area of southwest China. Hydrol. Processes.

[22] Ghimire C.P., Meerveld H.J., Zwartendijk B.W., Bruijnzeel L.A., Ravelona M., Lahitiana J., Lubczynski M.W. (2022). Vapour pressure deficit and solar radiation are the major drivers of transpiration in montane tropical secondary forests in eastern Madagascar. Agric. For. Meteorol..

[23] Nöjda P., Korpela M., Hari P., Rannik Ü., Sulkava M., Hollmén J., Mäkinen H. (2017). Effects of precipitation and temperature on the growth variation of Scots pine—A case study at two extreme sites in Finland. Dendrochronologia.

[24] Zhong Z., He B., Wang Y.P., Chen H.W., Chen D., Fu Y.H., Guo Y.C.L., Deng Y., Huang L., Yuan W. (2023). Disentangling the effects of vapor pressure deficit on northern terrestrial vegetation productivity. Sci. Adv..

[25] Cochard H. (2021). A new mechanism for tree mortality due to drought and heatwaves. Peer Community J..

[26] Maherali H., Pockman W.T., Jackson R.B. (2004). Adaptive variation in the vulnerability of woody plants to xylem cavitation. Ecology.

[27] Martin-StPaul N., Delzon S., Cochard H. (2017). Plant resistance to drought depends on timely stomatal closure. Ecol. Lett..

[28] Tyree M.T., Sperry J.S. (1988). Do woody plants operate near the point of catastrophic xylem dysfunction caused by dynamic water stress? Answers from a model. Plant Physiol..

[29] Tardieu F., Simonneau T. (1998). Variability among species of stomatal control under fluctuating soil water status and evaporative demand: Modelling isohydric and anisohydric behaviours. J. Exp. Bot..

[30] Klein T. (2014). The variability of stomatal sensitivity to leaf water potential across tree species indicates a continuum between isohydric and anisohydric behaviours. Funct. Ecol..

[31] McDowell N., Pockman W.T., Allen C.D., Breshears D.D., Cobb N., Kolb T., Plaut J., Sperry J., West A., Williams D.G. (2008). Mechanisms of plant survival and mortality during drought: Why do some plants survive while others succumb to drought?. New Phytol..

[32] Skelton R.P., West A.G., Dawson T.E. (2015). Predicting plant vulnerability to drought in biodiverse regions using functional traits. Proc. Natl. Acad. Sci. USA.

[33] Brodribb T.J., McAdam S.A. (2011). Passive origins of stomatal control in vascular plants. Science.

[34] Choat B., Brodribb T.J., Brodersen C.R., Duursma R.A., López R., Medlyn B.E. (2018). Triggers of tree mortality under drought. Nature.

[35] Nie Y., Chen H., Wang K., Ding Y. (2012). Rooting depth and water extraction of perennial grasses in a karst region. Plant Soil.

[36] Schwinning S. (2010). The ecohydrology of roots in rocks. Ecohydrology.

[37] Brodribb T.J., Holbrook N.M. (2003). Stomatal closure during leaf dehydration, correlation with other leaf physiological traits. Plant Physiol..

[38] Yang J., Chen H.S., Nie Y.P., Wang K.L. (2019). Dynamic variations in profile soil water on karst hillslopes in Southwest China. Catena.

